# CXCR3/CXCL10 interactions in the development of hypersensitivity pneumonitis

**DOI:** 10.1186/1465-9921-6-20

**Published:** 2005-02-22

**Authors:** Carlo Agostini, Fiorella Calabrese, Venerino Poletti, Guido Marcer, Monica Facco, Marta Miorin, Anna Cabrelle, Ilenia Baesso, Renato Zambello, Livio Trentin, Gianpietro Semenzato

**Affiliations:** 1 Padua University School of Medicine, Department of Clinical and Experimental Medicine, Clinical Immunology Branch, via Giustiniani 2, 35128 Padua. Italy; 2 Padua University School of Medicine, Department of Pathology, via Gabelli 61, 35121 Padua. Italy; 3 G.B: Morgani Hospital, Division of Pneumology, via Forlanini 34, 47100 Forlì, Italy; 4 Padua University School of Medicine, Department of Environmental Medicine and Public Healthy, via Giustiniani 2, 35128 Padua. Italy

## Abstract

**Background:**

Hypersensitivity pneumonitis (HP) is an interstitial lung disease caused by repeated inhalations of finely dispersed organic particles or low molecular weight chemicals. The disease is characterized by an alveolitis sustained by CD8(+) cytotoxic T lymphocytes, granuloma formation, and, whenever antigenic exposition continues, fibrosis. Although it is known that T-cell migration into the lungs is crucial in HP reaction, mechanisms implicated in this process remain undefined.

**Methods:**

Using flow cytometry, immunohistochemistry, confocal microscopy analysis and chemotaxis assays we evaluated whether CXCL10 and its receptor CXCR3 regulate the trafficking of CD8(+) T cells in HP lung.

**Results:**

Our data demonstrated that lymphocytes infiltrating lung biopsies are CD8 T cells which strongly stain for CXCR3. However, T cells accumulating in the BAL of HP were CXCR3(+)/IFNγ(+) Tc1 cells exhibiting a strong *in vitro *migratory capability in response to CXCL10. Alveolar macrophages expressed and secreted, in response to IFN-γ, definite levels of CXCL10 capable of inducing chemotaxis of the CXCR3(+) T-cell line. Interestingly, striking levels of CXCR3 ligands could be demonstrated in the fluid component of the BAL in individuals with HP.

**Conclusion:**

These data indicate that IFN-γ mediates the recruitment of lymphocytes into the lung via production of the chemokine CXCL10, resulting in Tc1-cell alveolitis and granuloma formation.

## Background

Hypersensitivity pneumonitis (HP) is an interstitial lung disease (ILD) caused by the inhalation of and sensitization to a variety of environmental organic antigens. The immune mediated nature of the disorder is testified to by the characteristic sequel of events taking place in the lung after antigenic inhalation: an acute pulmonary neutrophilia occurs early followed by an interstitial T-cell infiltration of CD8 T-cell showing a limited expression of the T-cell receptor [1].

A number of data point to chemokines as orchestrators of inflammatory disorders which are characterized by a massive accumulation of immunocompetent cells within affected organs, including the lung [2]. Chemokines, which can be divided into four groups based on the positioning of the cysteine residues in the mature protein [3-6], induce directional migration of immune cells through their interactions with G-protein coupled receptors. Three chemokines induced by IFN-γ, IFN-γ-inducible protein-10 (IP-10, CXCL10), monokine induced by IFN- (Mig/CXCL10), interferon-inducible T-cell α-chemoattractant (I-TAC/CXCL11) bind to the CXCR3 receptor molecule which is expressed by activated T lymphocytes and natural killer cells [7,8]. We have recently found that CXCR3 is expressed in vivo by CD4+ Th1 infiltrating the lung of patients with sarcoidosis and by T cells accumulating in the pulmonary parenchyma of lung-transplant recipients with rejection episodes [9,10], providing evidence that CXCR3 expression constitutes an important mechanism in the regulation of T-cell migration to the lung. Furthermore, recent data in the animal model suggest that CXCR3/CXCL9, CXCL10, CXCL11 interactions are central in the pathogenesis of hypersensitivity reactions to *Saccharopolyspora rectivirgula *(SR) and successive granuloma formation [11].

Using immunohistochemical studies of tissue sections and a flow cytometry evaluation of cells recovered from the bronchoalveolar lavage (BAL), we studied the role of CXCR3/CXCL10 interactions in the regulation of T-cell migration into the lung of patients with hypersensitivity pneumonitis. We have shown that CXCR3 is expressed by T cells accumulating in the lower respiratory tract of patients with this hypersensitivity disorder. In addition, we found that signalling of CXCR3 with CXCL10 induces the *in vitro *migration of CXCR3(+)T cells. The ligand CXCL10 can be detected in pulmonary macrophages and is released by these cells.

## Materials and Methods

### Study population

12 HP patients were included in the study (9 males and 3 females; mean age 38.3 ± 6.4 yr). The majority of the patients had farmer's lung disease (10 patients); 1 patient had bird fancier's lung, 1 patient had mushroom worker's lung. The following criteria for HP diagnosis were used: a) history of exposure to HP antigens, b) a symptomatic acute episode with chills, fever, cough, breathlessness 4 to 8 hours after exposure to specific antigens, c) radiological features (mainly diffuse reticular pattern) and/or a functional pattern of interstitial lung disease, and d) evidence of antibodies against S. rectivirgula in all except one case (bird fancier's lung). Each patient underwent bronchoscopy for transbronchial biopsy (TBB) and BAL analysis. BAL was performed according to the technical recommendations and guidelines for the standardization of BAL procedures [12]. Briefly, a total of 200 ml of saline solution was injected in 25-ml aliquots via fiberoptic bronchoscopy, with immediate vacuum aspiration after each aliquot. Immediately after the BAL, the fluid was filtered through gauze and the volume measured. A volume of 100-200 ml of BAL recovery and a sample of 50% of the instilled volume with a minimum of 50 ml was considered acceptable. The percentage of BAL recovery was 54.9% ± 4.2. Cells recovered from the BAL were washed 3 times with PBS, resuspended in endotoxin tested RPMI 1640 (Sigma Chemical Co., St. Louis, MO) supplemented with 20 mM HEPES and L-glutamine, 100 U/ml penicillin, 100 μg/ml streptomycin, and 10% FCS (ICN Flow, Costa Mesa, CA) and then counted. A standard morphological and immunologic analysis of BAL cellular components was performed and included cell recovery, differential count of macrophages, lymphocytes, neutrophils, and eosinophils, and flow cytometry analysis of the CD4/CD8 BAL T-cell ratio.

Five healthy controls were selected (3 men and 2 women; average age 37.3 ± 4.3 yr; 2 non-smoking healthy adults and 3 non-smoking subjects evaluated for complaints of cough without lung disease). They showed normal physical examinations, chest X-rays, lung function tests and BAL cell numbers.

### Purification of alveolar macrophages and T cells

Alveolar macrophages (AMs) and T cells were enriched from the BAL cell suspensions by rosetting with neuraminidase-treated SRBC followed by F/H gradient separations and residual CD3^+ ^lymphocytes were removed using high-gradient magnetic separation columns (Mini MACS, Miltenyi Biotec, Germany) [13]. Following this multistep selection procedure more than 95% of the above cells were viable, as judged by the trypan blue exclusion test. Staining with mAb showed that more than 99% of purified lymphocytes were CD3+ T cells.

### Monoclonal antibodies and cytokines

The commercially available conjugated or unconjugated mAbs used belonged to the Becton Dickinson and PharMingen series and included: CD3, CD4, CD8, isotype matched controls. Anti-IL-4 and anti-IFNγ mAbs were purchased from PharMingen (San Diego, CA). Purified rabbit anti-human CXCL10 polyclonal antibody (R&D Systems Inc, Minneapolis, MN) and anti-hCXCR3 mAb (R&D Systems Inc) were also used.

### Immunohistochemical analysis of CXCR3+ cells and CXCL10 producing cells

Open lung biopsies from 8 patients with clinical and histological diagnosis of hypersensitivity pneumonitis were studied by immunohistochemistry for the immunophenotype characterization of inflammatory cells and for CXCR3 and CXCL10 expression.

Immunohistochemistry for the characterization of inflammatory infiltrate was carried out using the following antibodies (Dako Glostrup, Denmark): CD45 (1:20), CD43 (1:40), CD45RO (1:100), CD20 (1:100), CD3 (1:50), CD68 (1:50), CD4 (1:100), and CD8 (1:100). The immunoreaction products were developed using the avidin-biotin-peroxidase complex method. Immunostaining for CXCR3 was performed as previously described. Briefly, after the microwave antigen retrieval procedure and neutralization of endogenous peroxidase activity, the slides were incubated with primary antibody for 1 hr in a humidified chamber at 37°C (anti-hCXCR3 mAb 1:100). Immunoreactivity was detected using biotinylated secondary antibodies incubated for 45 min followed by a 30 min incubation with avidin-peroxidase and visualized by a 7 min incubation with the use of 0.1% 3,3'-diaminobenzidene tetrahydrochloride as the chromogen. Parallel control slides were prepared either lacking primary antibody or lacking primary and secondary antibodies, or stained with normal sera to control for background reactivity. The intensity of antibody staining was classified in three groups: strong, weak, negative.

### Confocal microscopy for the identification of CXCR3+ cells

Paraffin sections were prepared for immunofluorescent labelling. Briefly, primary antibodies against CD3 and CXCR3 (1:100 diluted and 1:100 diluted in phosphate-buffered saline with 5 g/L bovine serum albumin and 1 g/L gelatine, respectively) and secondary antibodies (goat anti-mouse IgG and donkey anti-goat IgG) conjugated with TEXAS red or ALEXA 488 (Sigma) were used. Double labelling using both antibodies on the same section was performed. Primary antibodies and secondary antibodies were incubated for 1 h at room temperature. Nuclear staining was carried out with DAPI (Sigma) in PBS. Slides were stored at 4°C and analysed within 24 h. As a control, the primary antibody was omitted.

Immunofluorescence was evaluated with a confocal microscopy (Biorad 2100 Multiphoton; Hercules, CA), We used an argon laser at 488 nm in combination with a helium neon laser at 543 nm to excite the green (CD3) and red (CXCR3) fluorochromes simultaneously. Emitted fluorescence was detected with a 505–530 nm band pass filter for the green signal and a 560 nm long pass filter for the red signal. Images were analyzed using the Adobe Photoshop 7.0 program.

### Phenotypic evaluation of BAL cells

The frequency of BAL cells positive for the above reagents was determined by overlaying the flow cytometry histograms of the samples stained with the different reagents as previously reported [12]. Cells were scored using a FACScan^® ^analyzer (Becton Dickinson), and data were processed using the Macintosh CELLQuest software program (Becton Dickinson). The expression of cytoplasmic cytokine was evaluated following permeabilization of cell membranes using 1:2 diluted PermeaFix (Ortho, Raritan, NJ) for 40 min. After permeabilization procedures anti-IL-4, anti-IFN-γ and anti-CXCL10 antibodies were added. Since pulmonary cells bear cytoplasmic cytokines in a unimodal expression pattern, indicating that the entire cell population exhibits relatively homogeneous fluorescence, the percentage of positive cells does not represent the most accurate way of enumerating positive cells. Mean fluorescence intensity (MFI) was used to compare the positivity of these specific antigens on different cell populations. To evaluate whether the shift of the positive cell peak was statistically significant, the Kolmogorov-Smirnov test for analysis of histograms was used according to the Macintosh CELLQuest software user's guide (Becton Dickinson).

For immunofluorescence analysis, control IgG1 and IgG2a and IgG2b were obtained from Becton-Dickinson; control rat antiserum consisted of ascites containing an irrelevant rat IgG2b; control rabbit antiserum consisted of rabbit IgG (purified protein) purchased from Serotec (Serotec, U.K.); goat-anti-rabbit IgG and goat F(ab')2 anti-rat IgG were obtained from Immunotech (Marseille, France).

### Determination of IP-10/CXCL10 and Mig/CXCL9 mRNA levels

Each PCR product was analysed and quantitated by Bio-Rad's Image Analysis System Gel Doc using Quantity One software (Bio-Rad, Hercules, CA). Briefly, the images of the gels were acquired from the Gel Doc system densitometer and saved in digitised forms to perform volume analysis. The intensity of each band was differentiated by the intensity of the background, whose value was subtracted from each individual band and the resulting PCR product value was expressed in mm*mm*intensity of the pixels of the specific band in the gel.

### Generation of macrophage supernatants

To verify the ability of AMs to release CXCL10, AMs (1 × 10^6^/ml) were isolated from the BAL of HP patients, resuspended in RPMI medium and cultured for 24 hr in 24-well plates at 37°C in 5% CO_2_. In separate experiments AMs were stimulated with IFN-γ (100 U/ml), PMA (10 ng/ml) and LPS (10 μg/ml; Difco Lab., Detroit, MI). Following the incubation period, supernatants were harvested, filtered through a 0.45 μm Millipore filter and immediately stored at -80°C. At the end of the culture time AM viability was always greater than 95%. Chemotactic activity of supernatants was determined as reported below.

### Migration activity of pulmonary T cells in response to CXCLIO

T-cell migration was measured in a 48-well modified Boyden chamber (AC48 Neuro Probe Inc., USA). The chamber is made of two sections: different chemotactic stimuli were loaded in the bottom section while cells were added in the top compartment. Polyvinylpyrrolidone-free polycarbonate membranes with 3 to 5 μm pores (for lung T cells obtained from HP patients and the CXCR3+ and CXCR3- T-cell lines, respectively) (Osmonics, Livermore, CA) and coated with fibronectin were placed between the two chamber parts. Only the bottom face of filters was pretreated with fibronectin; the fibronectin pretreatment maximizes attachment of migrating cells to filters, avoiding the possibility that they may not adhere. Using this procedure in preliminary experiments we demonstrated that only a trivial number of cells may be recovered in the bottoms of the wells. To avoid the shedding of fibronectin, fibronectin-treated filters were extensively washed. In preliminary experiments, fibronectin-treated filters did not induce spontaneous chemotaxis in absence of chemokines.

To evaluate the migratory properties of pulmonary T lymphocytes rhIP-10/CXCL10 (200 ng/ml) were used. The CXCR3- and CXCR3+ cell lines (300-19, kindly provided by Dr. B. Moser, Theodor-Kocher Institute, University of Bern, Switzerland) were used as negative and positive controls. 30 μl of chemokines or control medium were added to the bottom wells, and 50 μl of 5.0 × 10^6 ^cells/ml T cells or CXCR3-/+ cells resuspended in RPMI 1640 were added to the top wells. The chamber was incubated at 37°C with 5% CO_2 _for 2 hrs. The membranes were then removed, washed with PBS on the upper side, fixed and stained with DiffQuik (Dade AG, Düdingen, Switzerland). Cells were counted in three fields per well at 800× magnification. All assays were performed in triplicate. In blocking experiments, cell suspensions were preincubated before chemotaxis assay for 30 min at 4°C with anti-human CXCR3 mAb at a concentration of 20 μg/ml.

### Chemotactic activity of the fluid component of BAL and macrophage supernatants

The CXCR3(-) and CXCR3(+) cell lines were also used to evaluate both the chemotactic activities of macrophage supernatants and the fluid component of BAL samples. Supernatants from cell cultures and the fluid components of BAL were obtained as reported above and used undiluted; different concentrations of CXCL10 were utilized as a positive control. Chemotactic assays were performed as reported above. In blocking experiments, anti-CXCL10 was added to the cell supernatants before chemotaxis assay at a concentration of 20 μg/ml.

### Statistical analysis

Data were analysed with the assistance of the Statistical Analysis System. Data are expressed as mean ± SD. Mean values were compared using the ANOVA test. A P value <0.05 was considered as significant.

## Results

### Immunohistochemical analysis of CXCR3 expression in lung biopsies

In all cases typical pathological examination showed features with poorly formed non-necrotizing granulomas and widespread thickening of the alveolar walls by a diffuse lymphocytic infiltrate. Pleural lymphoid aggregates were seen in a few cases and pleural lymphoid aggregates were seen in a few samples.

**Figure 1:**
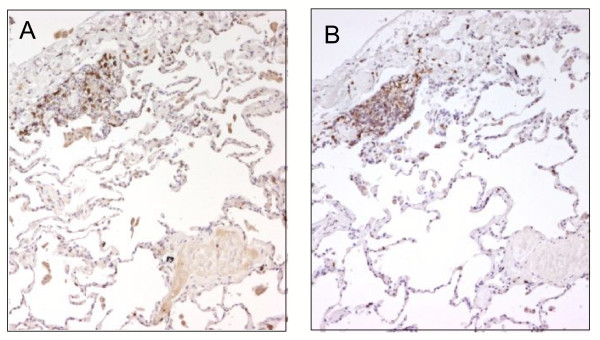
Immunohistochemistry for CD8 and CXCR3 in lung biopsy from HP patient. Most lymphocytes positive for CD8 (panel a) and CXCR3 (panel b) were clearly visible in a subpleural focus. Original Magnification × 25.

**Figure 2:**
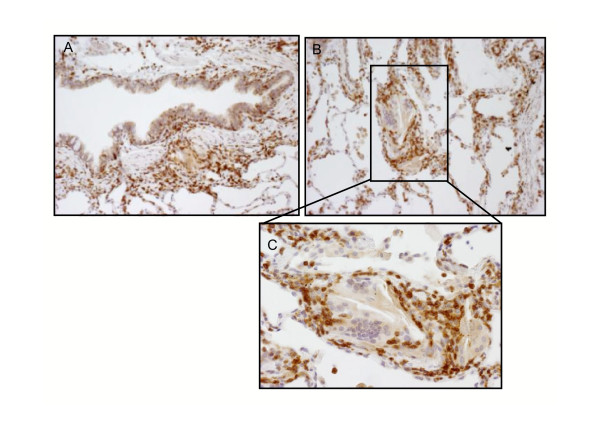
Immunohistochemistry for CXCR3 in lung biopsy from HP patient: positive marked lymphocytes while negative or weak staining macrophages were also seen in peribronchial space (panel A) and in the setting of non-necrotizing interstitial granuloma (panel B) (original magnification × 50). Note negative or weak staining of macrophages and giant cells forming the central core of the granuloma (inset panel C, original magnification × 100).

Diffuse interstitial lymphocytic infiltrates were characterized by an accumulation of T cells and a few B-lymphocytes. Sub-pleural and peri-bronchiolar nodules consisted mostly of T lymphocytes mainly represented by CD8 cytotoxic T lymphocytes which strongly stained for CXCR3 in all cases (Figure 1A and 1B). Marked CXCR3 immunostaining was also seen in peribronchial lymphocytic infiltrate and in the interstitial non-necrotising granuloma (Figure 2A and 2B) Both interstitial and intra-alveolar macrophages (CD 68 positive) showed weak or negative CXCR3 staining and multinucleated giant cells always stained negatively (Fig. 2C inset). Endothelial and epithelial cells close to more intense lymphocytic infiltrate were sometimes positively marked.

**Figure 3:**
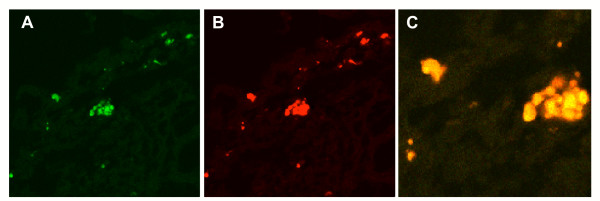
Immunofluorescence confocal laser scanning microscopy analysis shows the presence of CXCR3 (panel B, red) on CD3+ T cells (panel A, green). Panel C shows the overlay image of A and B in yellow (original magnification × 1000).

Confocal microscopy analysis of lung biopsies confirmed that lymphocyte infiltrates were formed by T cells coexpressing CXCR3 (Figure 3 panels A, B and C).

### Morphological and phenotypical features of cells obtained from the BAL

**Figure 4:**
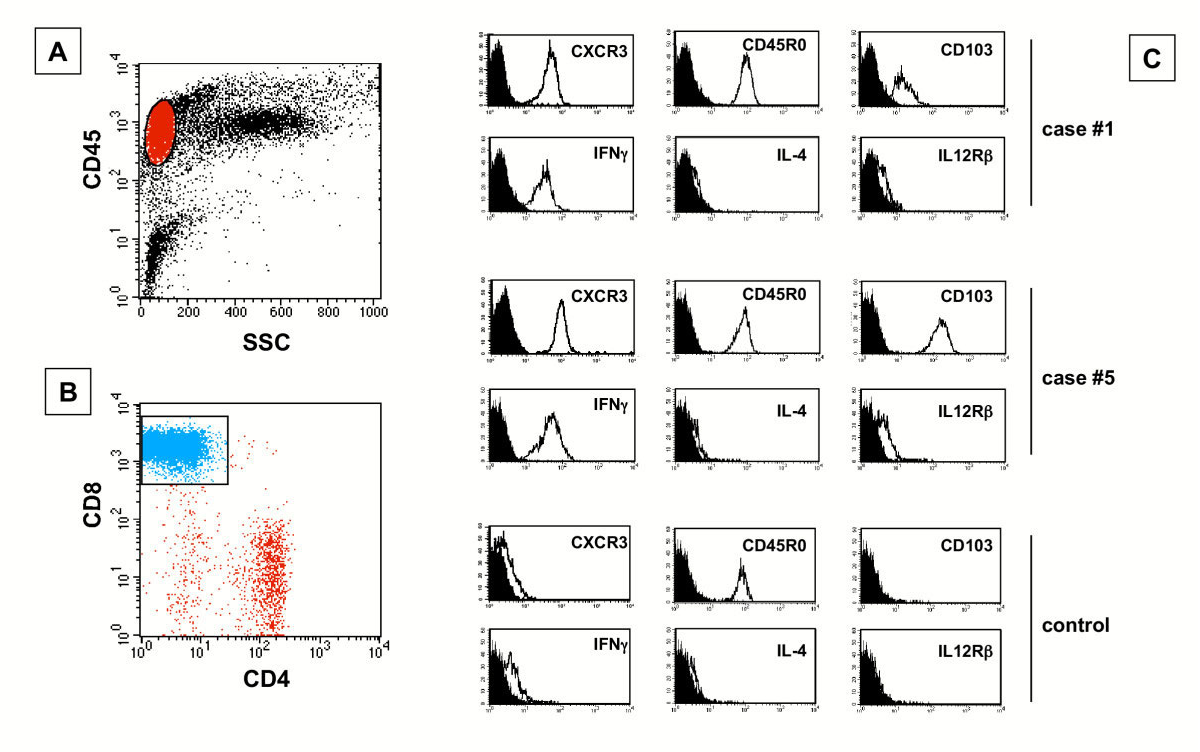
The flow cytometry profile of BAL T cells recovered from 2 representative patients with hypersensitivity pneumonitis and a control subject. BAL T cells were gated as shown in panel A. In patients with hypersensitivity pneumonitis the majority of lymphocytes were CD8(+) T cells (panel B). Panel C shows that BAL T cells from patients with hypersensitivity pneumonitis are CD45RO(+) T cells which express CXCR3, IFN-γ but not IL-4, or other activation markers including CD103 and IL12Rβ2.

Morphological and phenotypical features of cells obtained from the BAL of 12 patients with HP and 5 controls are reported in Table 1. All HP subjects showed a high intensity lymphocytic alveolitis sustained by CD8(+) Tc1 cells (Table 1 and Figure 4A and 4B). These cells were CXCR3(+) and bore IFN-γ but not IL-4 receptor (Figure 4C). Furthermore, pulmonary T cells expressed activatory molecules such as CD103 and IL-12β2 receptor (Figure 4C). The percentage and absolute number of BAL CXCR3(+) was significantly higher in HP patients with respect to control subjects (Table 2).

**Table 1:** Broncholaveolar findings in patients with hypersensitivity pneumonitis and controls

Study population	Cell Recovery	Lymphocytes	CD4 T cells		CD8 T cells	
				
	cells × 10^3^/ml	%	%	cells × 10^3^/ml	%	cells × 10^3^/ml
						
HP (n. 12)	351.9* ± 62.3	44.4** ± 8.1	25.6** ± 5.3	38.6*** ± 9.3	53.7** ± 6.3	83.4*** ± 8.5
						
Controls (n. 5)	138.6 ± 12.7	8.2 ± 2.2	48.3 ± 3.2	5.4 ± 0.9	23.7 ± 2.2	2.5 ± 0.3

Significance as follows: *p < 0.05; **p < 0.01; ***p < 0.001

**Table 2:** Expression of CXCR3 by CD8+ T cells and expression of IP-10/CXCL10 and Mig/CXCL9 mRNAs by alveolar macrophages from patients with hypersensitivity pneumonitis and controls*

Study population	CXCR3+/CD8+ T cells		IP-10/CXCL10	Mig/CXCL9
			
	%	cells × 10^3^/ml	mRNA levels*	mRNA levels
				
HP (n. 6)	51.7** ± 5.9	80.1*** ± 7.7	2.55** ± 0.14	2.25** ± 0.25
				
Controls (n. 4)	23.7 ± 2.2	2.5 ± 0.3	0.70 ± 0.05	0.48 ± 0.06

* Band intensity calculated as follows: mm × mm × pixel Significance as follows: **p < 0.01; ***p < 0.001

### CXCR3 mediates pulmonary T cell chemotaxis

**Figure 5:**
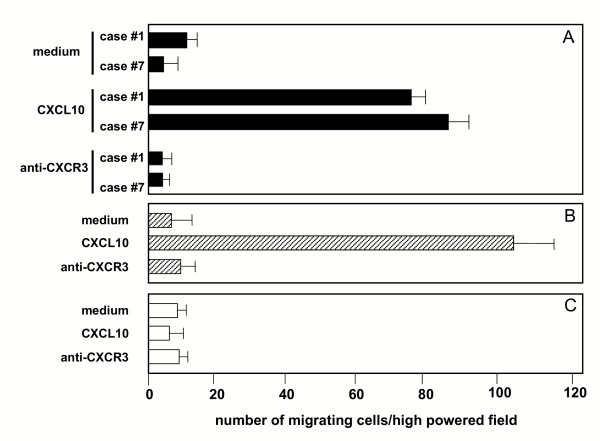
Chemotactic activity of CXCL10 on BAL CD8(+)/CXCR3(+)T cells highly purified from 2 representative patients with hypersensitivity pneumonitis. The assays were performed using a modified Boyden chamber in triplicate and data are given as mean ± SD. CXL10 shows significant chemotactic activity on BAL T cells (panels A) and the CXCR3(+) T-cell clone (panel B) but not on CXCR3(-) T-cell clone.

To define the biological activities of CXCR3, highly purified T cells obtained from the BALs of patients with HP were assessed for their migratory capabilities in response to different concentrations of CXCL10. The evaluation of the migratory potential of T lymphocytes obtained from the BAL of the controls was prevented by the low number of cells recovered. For this reason, the 300-19 T-cell lines expressing high levels of CXCR3 or not expressing CXCR3 were used as positive and negative controls respectively for the *in vitro *chemotaxis assay (Figure 5, panel B and C respectively).

As shown in panel A of Figure 4, the migratory capability of T lymphocytes of patients with HP is regulated by CXCR3. In fact, CXCR3+ lung T cells exhibited a strong, definite migration in response to CXCL10. To further verify the functional role of the CXCL10 receptor, CXCR3+ pulmonary T cells were preincubated with anti-CXCR3 neutralizing antibody: the blocking of the receptor determined a marked inhibition of CXCL10-induced chemotaxis (panel A). These data suggest that pulmonary T lymphocytes that sustain T-cell alveolitis in patients with HP express a functional CXCR3 receptor and actively migrate in response to CXCR3 ligands.

### Lung macrophages express CXCR3 ligands and release ligands showing chemotactic activity on CXCR3(+) cells

**Figure 6:**
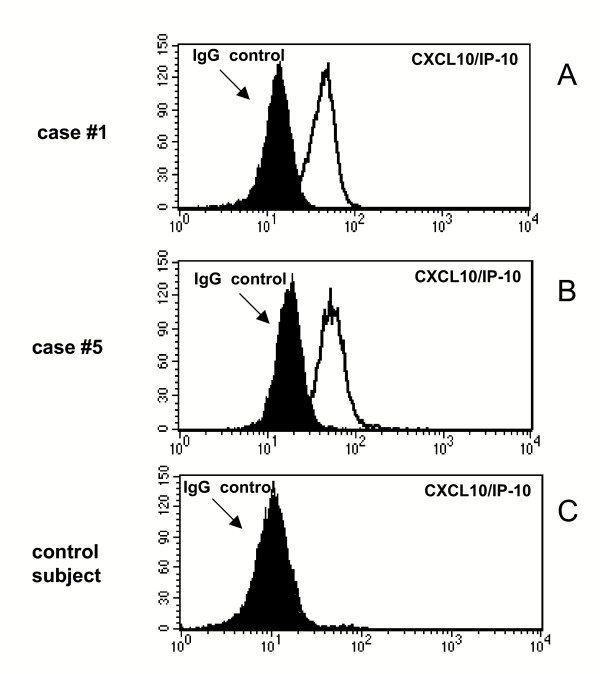
Flow cytometric analysis of CXCL10 expression by AMs infiltrating the lung of patients with hypersensitivity pneumonitis. Panels A-C shows the flow cytometry profile of AMs recovered from the BAL of 2 representative patients and a control subject. AMs from patients with hypersensitivity pneumonitis (panels A and B) but not control subject (panel C) express CXCL10.

In order to analyse whether CXCL10 is expressed *in vivo *by lung cells of patients with hypersensitivity pneumonitis, BAL cells were stained with a anti-CXCL10 antibody as described above. Flow cytrometric analysis (Figure 6, panels A and B) revealed that AMs of patients with HP express CXCL10; macrophages retrieved from control subjects lacked the CXCR3 ligand (panel C).

**Figure 7:**
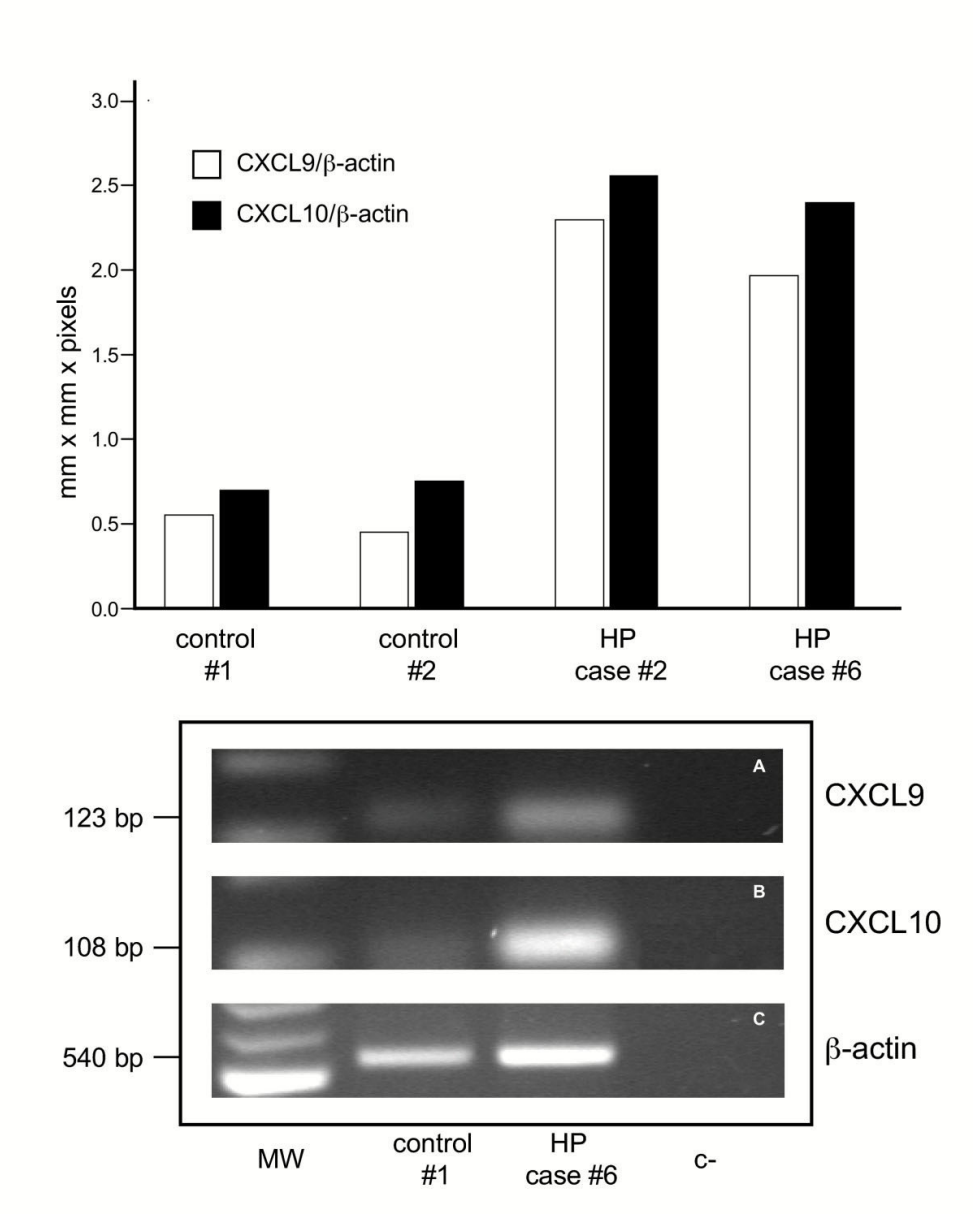
IP-10/CXCL10 and Mig/CXCL9 mRNA levels expressed by AMs recovered from the BAL of 2 representative patients and 2 control subject. AMs from patients with hypersensitivity pneumonitis express higher amounts of IP-10/CXCL10 and Mig/CXCL9 mRNA levels than control AMs.

Measurement of mRNA levels of the CXCR3 ligands demonstrated that unstimulated alveolar macrophages isolated from the BAL of HP subjects expressed increased mRNA levels of CXCL9 and CXCL10 with respect to macrophages obtained from control subjects (Table 2 and figure 7). Spearman Rank correlation coefficients between BAL T CD8(+)/CXCR3(+) T cell number and levels of CXCR3 ligands were also calculated. Interestingly, a positive correlation was demonstrated between mRNA levels of CXCL10 and CXCL9 and the absolute numbers of lung CD8(+)/CXCR3(+) T cells (r 0.815, p < 0.001 and r 0.825, p < 0.001, respectively).

Cell-free supernatants were obtained from 24-hour cultured AMs in the presence of IFN-γ and tested for their ability to induce T-cell migration. Supernatants obtained from AMs of patients with HP exerted chemotactic activity on the CXCR3(+) cell line; the CXCR3(-) cell line did not migrate in the presence of supernatants (data not shown). The addition of an anti-CXCL10 neutralizing antibody inhibited chemotactic activities of supernatants. The inhibitory activity shown by the neutralizing antibody was not complete, suggesting that other CXCR3 ligands (CXCL9 and CXCL11) are likely to be present in supernatants.

### CXCR3 ligands may be demonstrated in the fluid component of BAL

**Figure 8:**
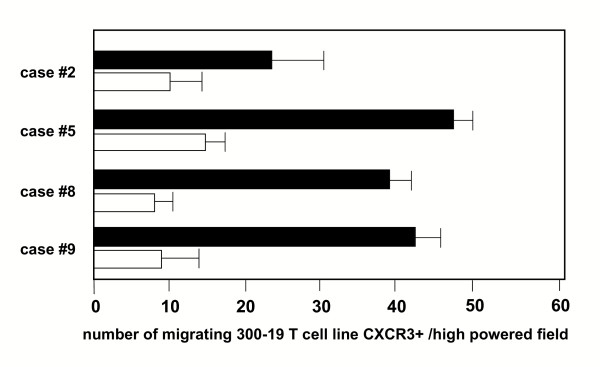
The fluid components of the BAL obtained from 4 patients with hypersensitivity patients exert chemotactic activity on the CXCR3(+) 300-19 T-cell line, indicating the presence of CXCR3 ligand(s). The migration was partially abrogated by an anti-CXCL10 neutralizing antibody (white bars). The assays were performed in triplicate, and data are given as mean ± SD.

To assess whether CXCR3 ligands are released *in vivo *in the lung microenvironment, the fluid component of BAL obtained from 10 HP patients was evaluated for chemotactic activity on CXCR3(+) cell lines (Figure 8). Measurable biological activity was demonstrated in 7 out of 10 patients with HP; this migration was partially abrogated by an anti-CXCL10 neutralizing antibody.

## Discussion

We have herein shown that CXCR3 expression represents a crucial mechanism which is involved in the recruitment of activated Tc1 cells in the pulmonary microenvironment of patients with HP. The continuous recruitment of CXCR3(+) T cells might play a role not only in the pathogenesis of T-cell alveolitis but also in favouring granuloma formation since T cells surrounding the macrophagic core of the HP granuloma expressed this chemokine receptor. This mechanism is likely to be shared by various ILDs since we and others have previously demonstrated the presence of a significant infiltrate of CXCR3(+) Th1 cells in other ILDs characterized by T cell alveolitis and granuloma formation, such as sarcoidosis and tuberculosis [13,14].

**Figure 9:**
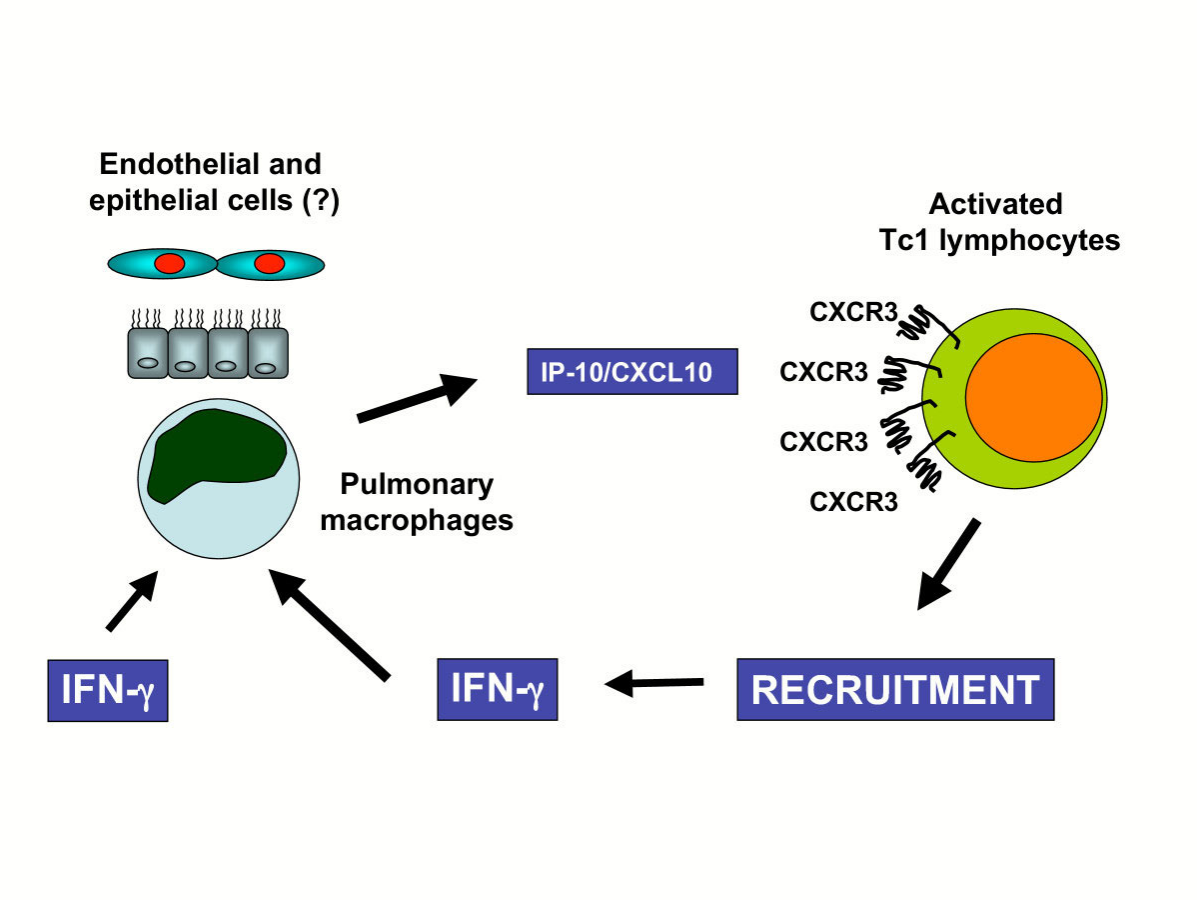
Putative scheme of the effect of the CXCR3/CXL10 interactions in the lung of hypersensitivity patients. As a consequence of the antigenic stimulation APC release IFN-γ. This cytokine stimulates AM to release CXCL10, a chemokine which favours the recruitments of activated CD8(+) /CXCR3(+) Tc1 lymphocytes. These cells, which release IFN-γ, in turn contribute to maintain the activation state of APC at sites of hypersensitivity reaction.

Our data provide definitive confirmation of the recent findings obtained in an animal model of IFN-γ-knockout (GKO) mice exposed to the particulate antigen *Saccharopolyspora rectivirgula *(SR) (i.e., the etiologic agent involved in the immunopathogenesis of HP reaction in the majority of our patients) [11]. While WT mice show the production of IP-10/CXCL10, Mig/CXCL9, and I-TAC/CXCL11 during the development of the classic HP reaction, GKO mice have reduced or no levels of IP-10/CXCL10, Mig/CXCL9 and I-TAC/CXCL11 in the lungs and reduced T-cell alveolitis in response to SR exposure. The present study suggests the role of CXCL10/CXCR3 and CXCL9/CXCR3 interactions in driving local CD8 immune responses to SR (Figure 9). A logical question is whether our data may have therapeutic implications. Because of the role of CXCR3 expression in the migration of T cells, strategies to block CXCL10 could in theory be proposed to prevent the development of HP reactions, particularly in subjects continuously exposed to inhaled antigens and thus at risk for the development of lung fibrosis. Further data are required to evaluate the *in vivo *role of IP-10/CXL10 in preventing or favouring pulmonary fibrosis in HP before proposing this strategy.

The relationship between CXCL10 release and the local production of other chemokines involved in HP immune response is another important aspect that deserves further investigation. In a murine model it has recently been shown that monocyte chemotactic protein-1 (MCP-1/CCL2) is increased in the BAL of mice challenged with SR [15]. Furthermore, Pardo et al. [16] have recently examined the expression of dendritic cell (DC)-derived CC chemokine 1 (CK1)/CCL18 in the lungs of patients with HP. CCL18 expression is significantly increased in lungs affected by HP, with higher levels in the subacute rather than in the chronic phase of the disease. Macrophages, dendritic cells, and alveolar epithelial cells are the main sources of CCL18 whose expression is induced by various profibrogenic cytokines including IL-4, IL-10, and IL-13. Interestingly, a direct correlation between the levels of tissue CCL18 and the number of lymphocytes has been demonstrated in the bronchoalveolar lavage fluids.

Thus, our findings and the data of Pardo et al. [16] suggest that chemokines ordinarily induced by profibrogenic cytokines (CCL18) and chemokines induced by antifibrotic agents (IFN-γ and CXCL10) can be demonstrated in the lung of HP patients with T cell alveolitis. Whether the presence of the two chemokines is concomitant and there are common molecular mechanisms involved in the CC and CXC chemokine release is unknown. Given the heterogeneous pattern of pulmonary infiltrate during different phases of the disease, a full understanding of the interdependence of the local hyperproduction of chemoattractant molecules may help to clarify the pathogenesis of HP. In this context, it is also mandatory to investigate the production of CC chemokines in relationship with CXCL10 release in individuals with acute, subacute and chronic HP. For instance, it is known that the combination of RANTES/CCL5 and CXCL10 but not other chemokines (MIP-1α/CCL3, MIP-1β/CCL4, Mig/CXCL9, and ITAC/CXCL11) markedly increases T-cell recruitment [17]. Since there are data indicating that circulating, antigen-reactive, memory T cells, generated by previous sensitization to organic antigens, migrate into lung parenchyma in response to chemokines such as RANTES [18], it is possible that the interplay of CXCL10 with CCL5 may serve to finely tune inflammatory responses *in vivo *in HP lungs.

## Conclusion

Our findings clearly indicate the effects of CXCR3/CXCL10 interactions on hypersensitivity reaction to SR antigens. Considering the importance of CD8 T cells in mediating granuloma formation and lung damage, further studies are needed in animal models to explore the therapeutic potential of CXCR3 antagonists with the ultimate goal of offering new clues for immune intervention in subjects continuously exposed to inhaled antigens and thus at risk of developing HP-related lung fibrosis.

## List of abbreviations

AM: alveolar macrophage; BAL: bronchoalveolar lavage; CXCR3: Receptor 3 for CXC chemokines; HP: Hypersensitivity pneumonitis; IP10/CXCL10: IFN-γ-inducible protein-10; I-TAC/CXCL11: interferon-inducible T-cell αlfa-chemoattractant; Mig/CXCL10: monokine induced by IFN-γ; TBB: transbronchial biopsy.

## Authors' contributions

CA: planned the experimental design, coordinated the research group and drafted the manuscript

FC: participated in the study design and in immuohiostochemical analysis

VP: participated in the study design and in the bronchoalveolar lavage execution

GM: participated in the study design and in the clinical evaluation of the patients

MF: participated in the study design and performed migration assays studies

MM: participated in the study design and performed cellular studies

AC: participated in the study design and performed flow cytometry studies

IB: participated in the study design and performed flow cytometry studies

RZ: participated in the study design and helped the draft of the manuscript

LT: participated in the study design and helped the draft of the manuscript

GS: participated in the study design and coordination of the research group
